# What Factors Might Have Led to the Emergence of Ebola in West Africa?

**DOI:** 10.1371/journal.pntd.0003652

**Published:** 2015-06-04

**Authors:** Kathleen A. Alexander, Claire E. Sanderson, Madav Marathe, Bryan L. Lewis, Caitlin M. Rivers, Jeffrey Shaman, John M. Drake, Eric Lofgren, Virginia M. Dato, Marisa C. Eisenberg, Stephen Eubank

**Affiliations:** 1 Department of Fisheries and Wildlife Conservation, Virginia Tech, Blacksburg, Virginia, United States of America; 2 Department of Computer Science, Virginia Tech, Blacksburg, Virginia, United States of America; 3 Network Dynamics and Simulation Science Laboratory, Virginia Bioinformatics Institute, Virginia Tech, Blacksburg, Virginia, United States of America; 4 Department of Environmental Health Sciences, Mailman School of Public Health, Columbia University, New York, New York, United States of America; 5 Odum School of Ecology, University of Georgia, Athens, Georgia, United States of America; 6 Department of Biomedical Informatics, School of Medicine, University of Pittsburgh, Pittsburgh, Pennsylvania, United States of America; 7 Departments of Epidemiology and Mathematics, University of Michigan, Ann Arbor, Michigan, United States of America; Common Heritage Foundation, NIGERIA

## Abstract

An Ebola outbreak of unprecedented scope emerged in West Africa in December 2013 and presently continues unabated in the countries of Guinea, Sierra Leone, and Liberia. Ebola is not new to Africa, and outbreaks have been confirmed as far back as 1976. The current West African Ebola outbreak is the largest ever recorded and differs dramatically from prior outbreaks in its duration, number of people affected, and geographic extent. The emergence of this deadly disease in West Africa invites many questions, foremost among these: why now, and why in West Africa? Here, we review the sociological, ecological, and environmental drivers that might have influenced the emergence of Ebola in this region of Africa and its spread throughout the region. Containment of the West African Ebola outbreak is the most pressing, immediate need. A comprehensive assessment of the drivers of Ebola emergence and sustained human-to-human transmission is also needed in order to prepare other countries for importation or emergence of this disease. Such assessment includes identification of country-level protocols and interagency policies for outbreak detection and rapid response, increased understanding of cultural and traditional risk factors within and between nations, delivery of culturally embedded public health education, and regional coordination and collaboration, particularly with governments and health ministries throughout Africa. Public health education is also urgently needed in countries outside of Africa in order to ensure that risk is properly understood and public concerns do not escalate unnecessarily. To prevent future outbreaks, coordinated, multiscale, early warning systems should be developed that make full use of these integrated assessments, partner with local communities in high-risk areas, and provide clearly defined response recommendations specific to the needs of each community.

## Introduction

**Fig 1:**
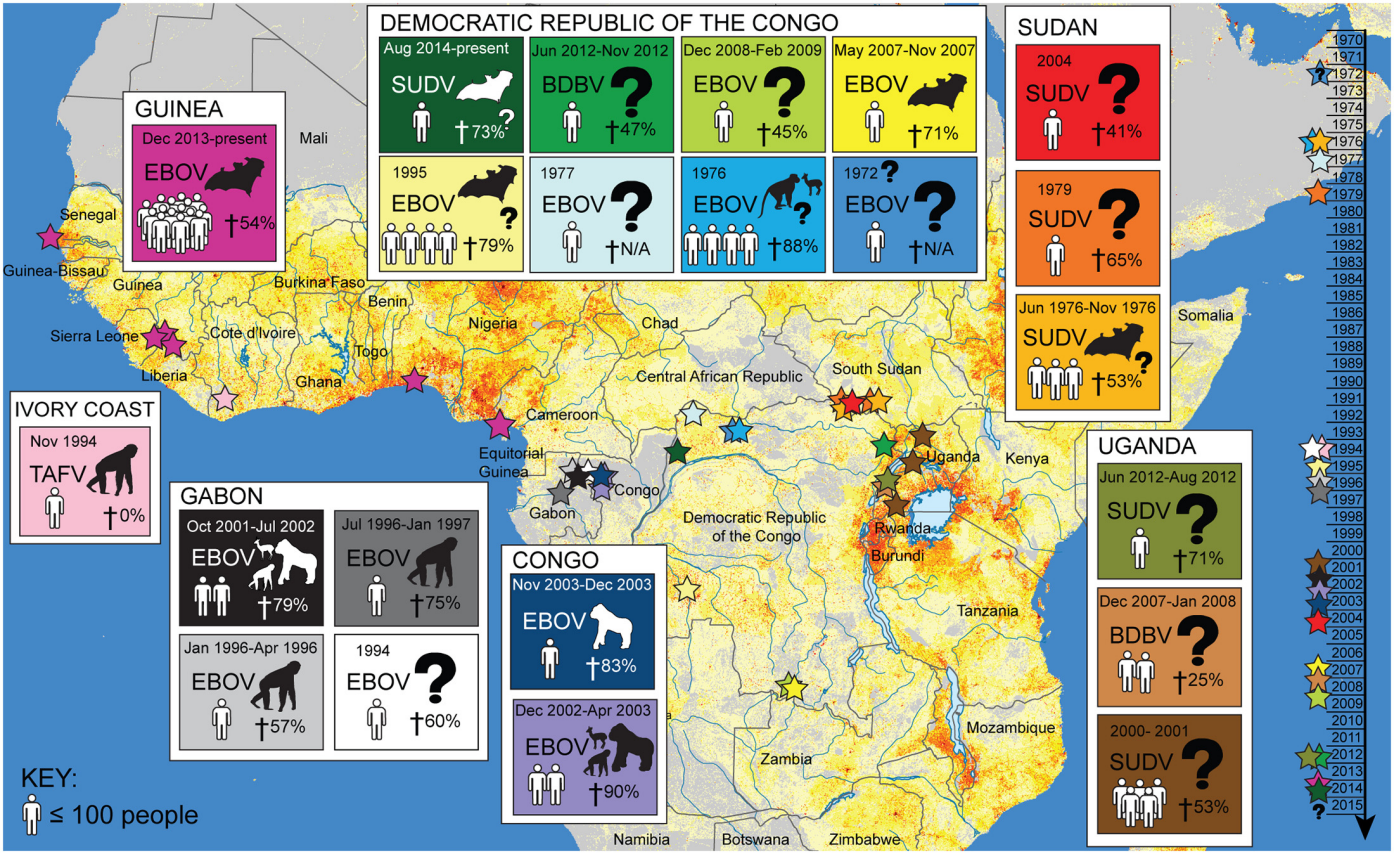
Map of Ebola outbreaks in Africa. The outbreak in West Africa is unprecedented in its scope and duration, occurring for the first time in urban centers. Historically, Ebola viral outbreaks (stars, timeline right) occurred sporadically, limited largely to Central African rural areas where the human population (grey to red gradient stippling [5,6]) has been low or more remote from areas of high population density. It is uncertain how frequent Ebola outbreaks will be in the future, given the identification of wildlife spillover potential in West Africa and the increasingly concentrated human populations in this region.

On December 6, 2013, the world’s largest Ebola epidemic began when a two-year-old in Guéckédou, Guinea, a small village bordering Sierra Leone and Liberia, became infected (Fig 1) [1,2]. This is the first documented Ebola outbreak outside Central Africa and is unique in its size, duration, and spatial extent. The circulating virus has been identified as the Zaire ebolavirus (EBOV), a strain previously found in only three Central African countries: the Democratic Republic of the Congo (DRC), Republic of the Congo, and Gabon (Fig 1) [3]. The public health impact of the current Ebola epidemic in West Africa has been far greater than case counts. Massive indirect effects on already-weakened public services have occurred, including significant crippling of the health sector, which has increased the impacts of other endemic diseases and the associated mortality [3]. Substantial economic loss and social disruption will have a sustained impact on the region that will far outlive the actual epidemic [4]. One paper, published in 2011, argued that Ebola would never become a significant public health threat in Africa [4]; clearly, the threat of Ebola has been underestimated. The emergence of Ebola in West Africa invites many questions—most of which remain unresolved—notably: why now, and why in West Africa? Advancing our understanding of this outbreak remains critical to present health care interventions as well as the prevention of further outbreaks. Here, we review the sociological, ecological, and environmental drivers that could have influenced the emergence of EBOV in West Africa at this time and in this manner. Given these factors, we explore the lessons of this outbreak and evaluate how we might manage future threats from Ebola across the complex urban and rural landscapes that define modern Africa.

## Ebola in Africa

**Fig 2:**
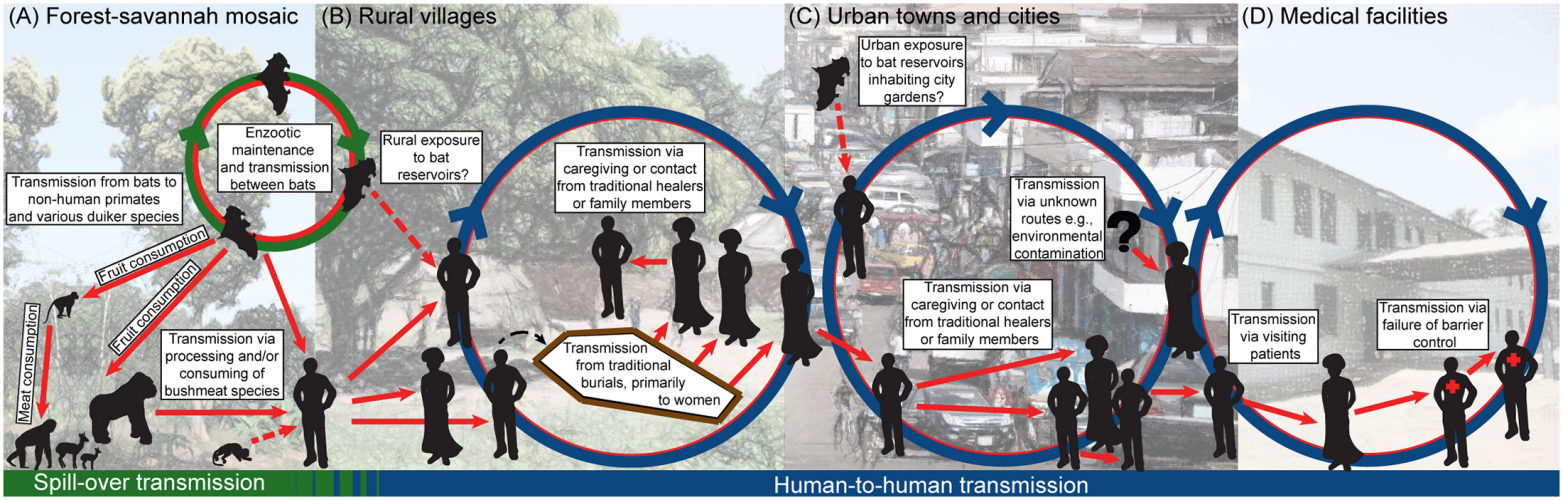
Schematic of virus spillover from wildlife and human-to-human transmission. Pathogen spillover to humans is typically associated with the use of bushmeat and direct contact with tissues and/or bodily fluids through handling and eating of infected animals (A), e.g., duiker, primates, or fruit bats [13]. Predation and consumption of a red colobus monkey by chimpanzees has also been linked to an outbreak of Ebola among chimpanzees and one researcher in Côte d'Ivoire [14]. Ingestion of fruit contaminated with Ebola-infected bat saliva or feces may be another mechanism by which bats might infect other involved wildlife species (e.g., duiker, nonhuman primates) or even humans. Human-to-human transmission has been associated with traditional burial practices, caregiving, or some other form of direct physical contact with infected individuals or bodily fluids [15]. Transmission dynamics in high-density urban centers (C) will differ importantly from rural villages (B), influencing outbreak progression and control efforts. Transmission in the hospital setting is largely associated with failures in infection control procedures and standard barrier precautions (D), many of which are related to inadequate staffing, infrastructure, and financing of health care systems [16,17].

Ebola hemorrhagic fever is an emerging zoonotic viral disease that historically has occurred in rural areas of Central Africa, with isolated cases identified elsewhere (Fig 1 and Table 1). The Ebola virus was first identified in humans in southern Sudan in 1976 [7], but likely occurred as early as 1972 in Tandala, DRC [8]. The virus causes severe morbidity and high mortality in humans and wildlife [9]. Humans typically are infected with Ebola either through contact with bodily fluids of infected animals or humans, or through consumption of bushmeat, caring for patients, or preparing the deceased for burial (Fig 2) [10]. EBOV can be found in a number of human secretions during the acute phase of infection, such as saliva, feces, semen, breast milk, tears, nasal blood, and skin [11]. Presently, there is no vaccine or other therapeutic interventions beyond supportive care, although promising pharmaceutical options are on the horizon, including vaccines [12].

**Table 1:** Ebola Outbreaks in Africa.

Date	Location of first case	Countries affected	Strain	Number of human cases	Number of human deaths	Mortality	Reservoir
Aug 2014–present	Equator Province, DRC	DRC	SUDV	67	49	73%	Possibly fruit bats
Dec 2013–present	Guéckédou, Guinea	Guinea, Liberia, Sierra Leone, Nigeria, Senegal	EBOV	5481*	2946	54%	Fruit bats
Jun 2012–Nov 2012	Province Orientale, DRC	DRC	BDBV	77	36	47%	Unknown, although bushmeat likely
Jun 2012–Aug 2012	Kibaale District, Uganda	Uganda	SUDV	24	17	71%	Unknown
Dec 2008–Feb 2009	Kasai-Occidental Province, DRC	DRC	EBOV	32	14	45%	Unknown
Dec 2007–Jan 2008	Bundibugyo District, Uganda	Uganda	BDBV	149	37	25%	Unknown
May 2007–Nov 2007	Kasai-Occidental Province, DRC	DRC	EBOV	264	187	71%	Fruit bats
Apr 2004–Aug 2004	Yambio county, Sudan	Sudan	SUDV	17	7	41%	Unknown
Nov 2003–Dec 2003	Mbono District, Congo	Congo	EBOV	35	29	83%	Gorilla
Dec 2002–Apr 2003	Mbono and Kéllé Districts, Congo	Congo	EBOV	143	128	90%	Possibly duiker, chimpanzee, and gorilla
Oct 2001–Jul 2002	Makokou and Mékouka, Gabon Border	Gabon, Congo	EBOV	122	96	79%	Possibly duiker, chimpanzee, and gorilla
2000–2001	Gulu, Masinsi, and Mbarara districts, Uganda	Uganda	SUDV	425	224	53%	Unknown
Jul 1996–Jan 1997	Booué, Gabon	Gabon	EBOV	60	45	75%	Chimpanzee
Oct 1996	Johannesburg, South Africa	South Africa	EBOV	2	1	N/A	Human travelling from Gabon
Jan 1996–Apr 1996	Mayibout, Gabon	Gabon	EBOV	37	21	57%	Chimpanzee
1995	Kikwit, DRC	DRC	EBOV	315	250	79%	Possibly fruit bats
Nov 1994	Taï National Park, Ivory Coast	Ivory Coast	TAFV	1	0	0%	Chimpanzee
1994	Mékouka, Gabon	Gabon	EBOV	52	31	60%	Gorilla
1979	Nzara and Maridi, Sudan	Sudan	SUDV	34	22	65%	Unknown
1977	Tandala, DRC	DRC	EBOV	1	1	N/A	Unknown
Aug 1976	Yambuku, DRC	DRC	EBOV	318	280	88%	Possibly antelope or monkey
Jun 1976–Nov 1976	Nzara and Maridi, Sudan	Sudan	SUDV	284	151	53%	Possibly fruit bats

Ebola outbreaks have been confirmed in Africa since 1976 [6]. Since then, four different strains of Ebola have emerged in Central and West Africa, from varying presumptive wildlife sources. These strains include Zaire ebolavirus (EBOV), Sudan ebolavirus (SUDV), Bundibugyo ebolavirus (BDBV) and Taï Forest ebolavirus (TAFV).

*Number of laboratory-confirmed cases only.

Virus invasion in humans appears to occur through mucosal surfaces, breaks and abrasions in the skin, or parenteral introduction (reviewed in [18]). Route of exposure is important in determining the course of disease. During the 1976 outbreak in the DRC, the incubation period in humans exposed to EBOV through injection (in association with unsterilized needle reuse) was shorter than individuals exposed through known contacts (5–9 days, in respect of virus strains circulating in that outbreak [19]). Case fatality rates also differed by exposure route, with 100% mortality among those exposed through injection (85 out of 85) and 80% among cases with known contact (119 of 149). In laboratory studies of EBOV infection in nonhuman primates, the disease course was more rapid with exposure through intramuscular or intraperitoneal injection than through aerosol droplets [20]. Aerosol transmission has been identified only in laboratory settings [21] and is thought to be rare or absent in natural outbreaks [18]. Oral and conjunctival EBOV exposure was found to be extremely lethal in experimentally infected rhesus macaques [22]. Additionally, organs from laboratory-infected, nonhuman primates had extremely high infectivity titers (5.5–8.6 log10 pfu/g, [20]), indicating that exposure to high infectious doses might occur with consumption.

### West African outbreak 2014

**Fig 3:**
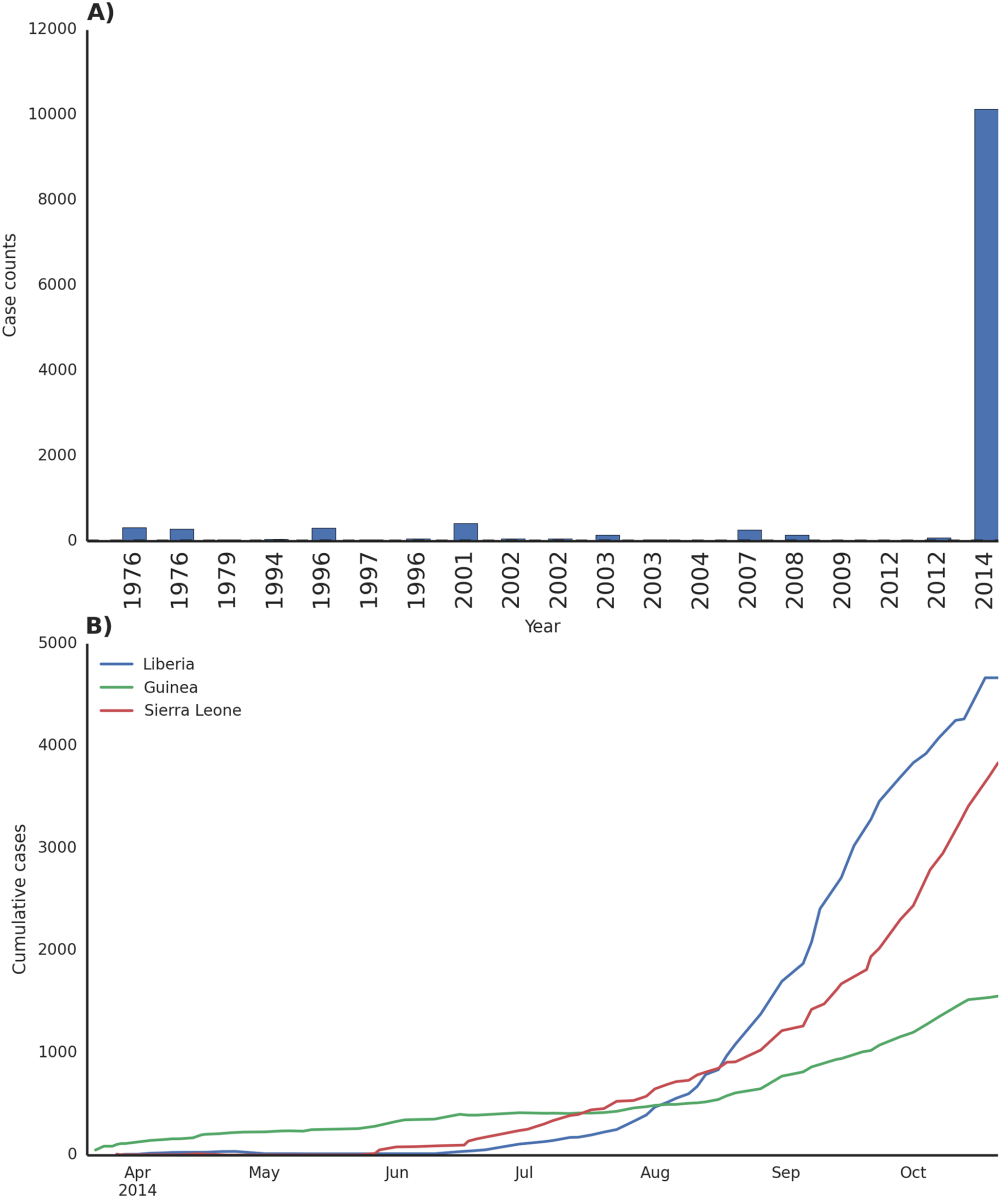
Case counts of historical Ebola outbreaks and the current outbreak in West Africa [24]. A) The 2014 West Africa outbreak eclipses all previous known outbreaks, with more cases and deaths than the other events combined. B) Cumulative case counts in Liberia, Guinea, and Sierra Leone demonstrate widespread transmission. Presently, Liberia is experiencing intense growth of the disease outbreak, with dozens of new cases each day.

The World Health Organization (WHO) designated the West African outbreak as a Public Health Emergency of International Concern (PHEIC) on August 8, 2014 [23]. As of October 25, the WHO reported 10,141 cases and 4,922 deaths, making this ongoing outbreak several times larger than all previous Ebola outbreaks combined (Fig 3A) [24]. Even so, those numbers may be a drastic underestimate of the true case burden. In late August, the WHO estimates the true prevalence to be two to four times higher than the reported figures [25]. The outbreak is concentrated in the capitals of Guinea, Liberia, and Sierra Leone, although cases have occurred in nearly all regions of these countries.

**Fig 4:**
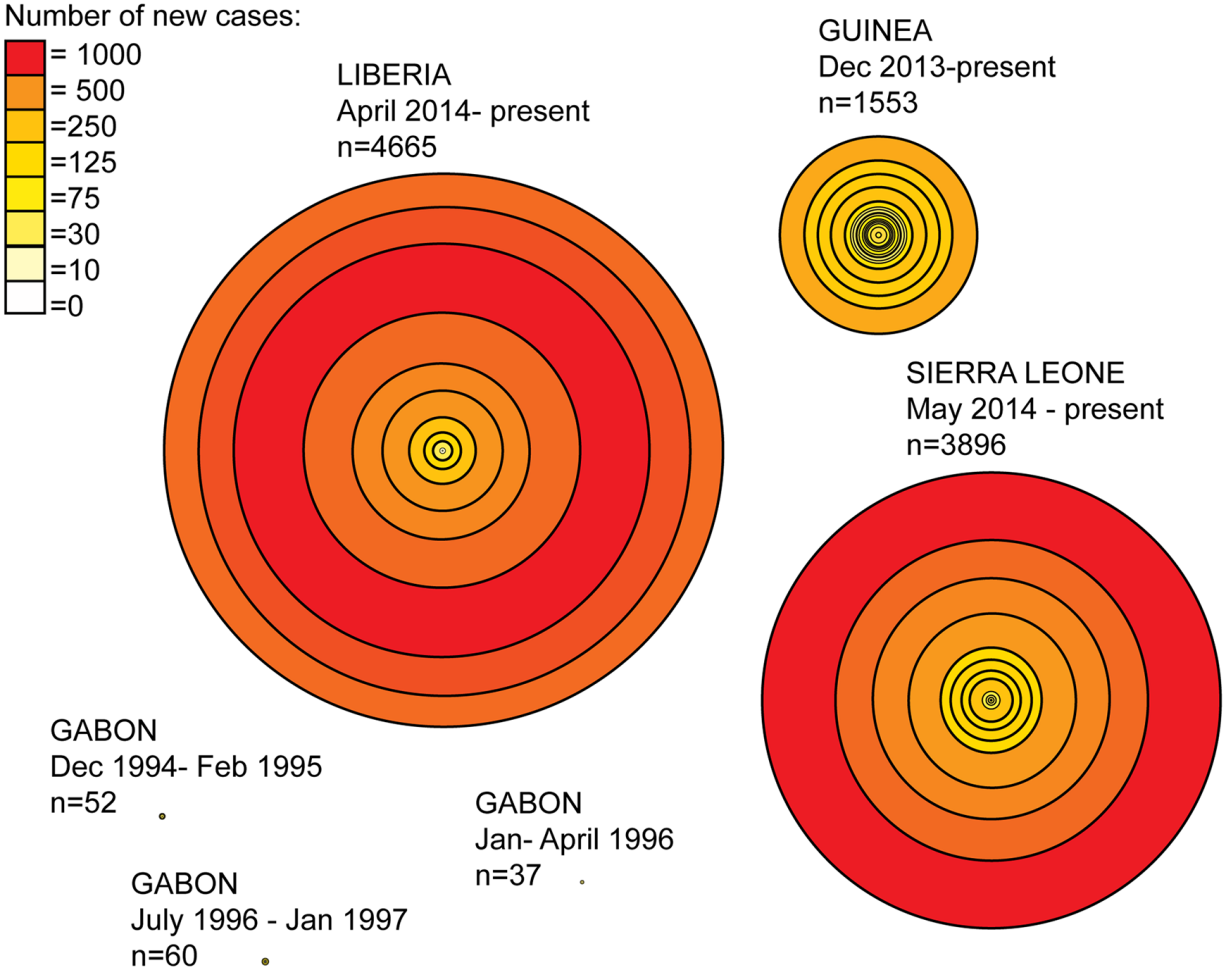
West Africa Ebola case counts at biweekly intervals. An assessment of EBOV outbreaks in which circles identify two-week intervals in outbreak progression, and distance between the circle lines is equivalent to the number of cases affected during that respective time period. The graphic highlights the important differences in outbreak duration and case counts not only between West Africa and Central Africa EBOV epidemics but also by country within the outbreak region in West Africa itself. Liberia clearly has had the largest number of cases over the shortest duration. This reflects, in part, the movement of the outbreak into the high-density urban center—the capital city Monrovia—and the intense growth of the outbreak from that point.

Presently, there is little evidence of epidemic control in West Africa (Table 2) [26,27]. The recently developed model EbolaResponse provides a tool to estimate the potential increase in Ebola cases (available at http://dx.doi.org/10.15620/cdc.24900) [27]. It was predicted that if there were no significant changes made in outbreak management, the total number of Ebola cases could reach 21,000 in Liberia and Sierra Leone by the end of September 2014 [27]. This forecast included a correction for estimates of suspected under-reporting [27]. Despite this estimate being significantly less than the current reported number of cases [24], Ebola transmission is still widespread and intense in the West Africa region (Figs 3B and 4) [24,28]. EBOV infections have occurred beyond these core outbreak countries in Nigeria, Senegal, and Mali [24]. Model simulations using mobility and airline data indicate the threat of international dissemination beyond the Africa region through air travel is limited [29], despite secondary spread occurring in Spain and the United States [24]. Current intervention focus is on the rapid increase in treatment facilities and capacity to isolate infected patients in the affected countries in order to reduce Ebola transmission within the population [27,30].

**Table 2:** Epidemiological characteristics of the 2014 West African Ebola outbreak.

Summary of Ebola outbreak characteristics in West Africa		
December–September 2014 [26]		
Term	Definition	Current estimates
Reproductive number (R_0_):	*Number of healthy people one sick individual infects over the course of his/her illness*.	Guinea: 1.71
		Liberia: 1.83
		Sierra Leone: 2.02
Serial interval:	*Time between consecutive people falling ill in a chain of transmission*.	15.3 days
Incubation period:	*Amount of time passed between a person becoming exposed to Ebola and when they start to show symptoms of the disease*.	11.4 days
Doubling time:	*Time taken for the number of sick individuals to double*.	Guinea: 15.7 days
		Liberia: 23.6 days
		Sierra Leone: 30.2 days
Confirmed case fatality rate:	*Number of people who die of confirmed Ebola infection*.	Guinea: 70.7%
		Liberia: 72.3%
		Sierra Leone 69.0%
Unconfirmed case fatality rate:	*Number of people who die with suspected but not confirmed Ebola infection*.	Guinea: 13%
		Liberia: 58%
		Sierra Leone: 35%

Concerns of epidemic spread beyond Africa to places such as the US have occupied the public’s attention and now have become important topics of concern and fear. A recent national survey found 39% of adult respondents believed there would be a large outbreak of Ebola within the US in the next 12 months [31]. Respondents with lower levels of education were more likely to express these views [24]. Unlike other viruses, such as influenza, that are airborne and can be transmitted through casual contact [32], Ebola requires direct physical contact with bodily fluids from a clinically ill person [15]. Accordingly, the only two cases of secondary transmission to occur in the US were associated with nursing staff and care of an Ebola patient [33]. Since these events, CDC guidelines and other safety protocols have been revised and strengthened [34]. More aggressive approaches, such as mandatory quarantine for returning medical personnel, have also been employed in some states [35], creating concerns that unnecessary fear and precaution may impact medical personnel and willingness to assist in the West African outbreak [36]. Public health education is urgently needed not only in West Africa but also within the US.

Full genome sequencing of EBOV isolates from the Sierra Leone outbreak region from May–August of 2014 (*n* = 99) [37] and previous molecular sequencing studies of a limited number of Guinean EBOV cases [10] provided similar results, suggesting that the West African outbreak arose from a single spillover event from a wildlife reservoir with subsequent sustained human-to-human transmission. However, important spatial and temporal limitations existed in sample collection in these studies. Accordingly, these conclusions can only be applied to the region of the outbreak area assessed. Additional studies will be necessary to fully understand EBOV transmission dynamics and the role of virus spillover from animal hosts.

### Is the virus in the West African outbreak changing?

Phylogenetic analyses indicate that the virus strain in the current outbreak likely originated from Central Africa around 2004 [37]. In Sierra Leone, the outbreak is believed to have started from the introduction of two genetically different viruses from Guinea, where people were attending a funeral [37]. These two viruses diverged in Guinea in late April, before they were discovered in Sierra Leone a month later [37]. These sequencing efforts identified 396 genetic mutations that have occurred over time, including 50 nonsynonymous mutations since separation from the Central African lineage. During this current outbreak, the frequency of nucleotide substitution rates has been approximately two times higher than that observed across all previous Ebola outbreaks from which sequence data were available. Substitutions have been more commonly nonsynonymous [37], which change the amino acid sequence of the virus and could potentially be correlated with phenotypic changes that might influence outbreak dynamics and virus behavior. While more research is required to understand the effect of increased nonsynonymous mutation rates in the West Africa EBOV virus population, the sustained nature of the outbreak increases the opportunity for further change in the virus, with uncertain consequences [37]. However, as yet, similarity in outbreak characteristics (including R_0_, symptoms, incubation time, serial time) between the West Africa 2014 outbreak and previous Ebola outbreaks suggests that there has not been any significant change in the virus affecting transmissibility (Table 2) [26]. Rather, outbreak progression appears to be more strongly influenced by the urban setting of the outbreak and other socioeconomic features.

### The Democratic Republic of Congo 2014

**Fig 5:**
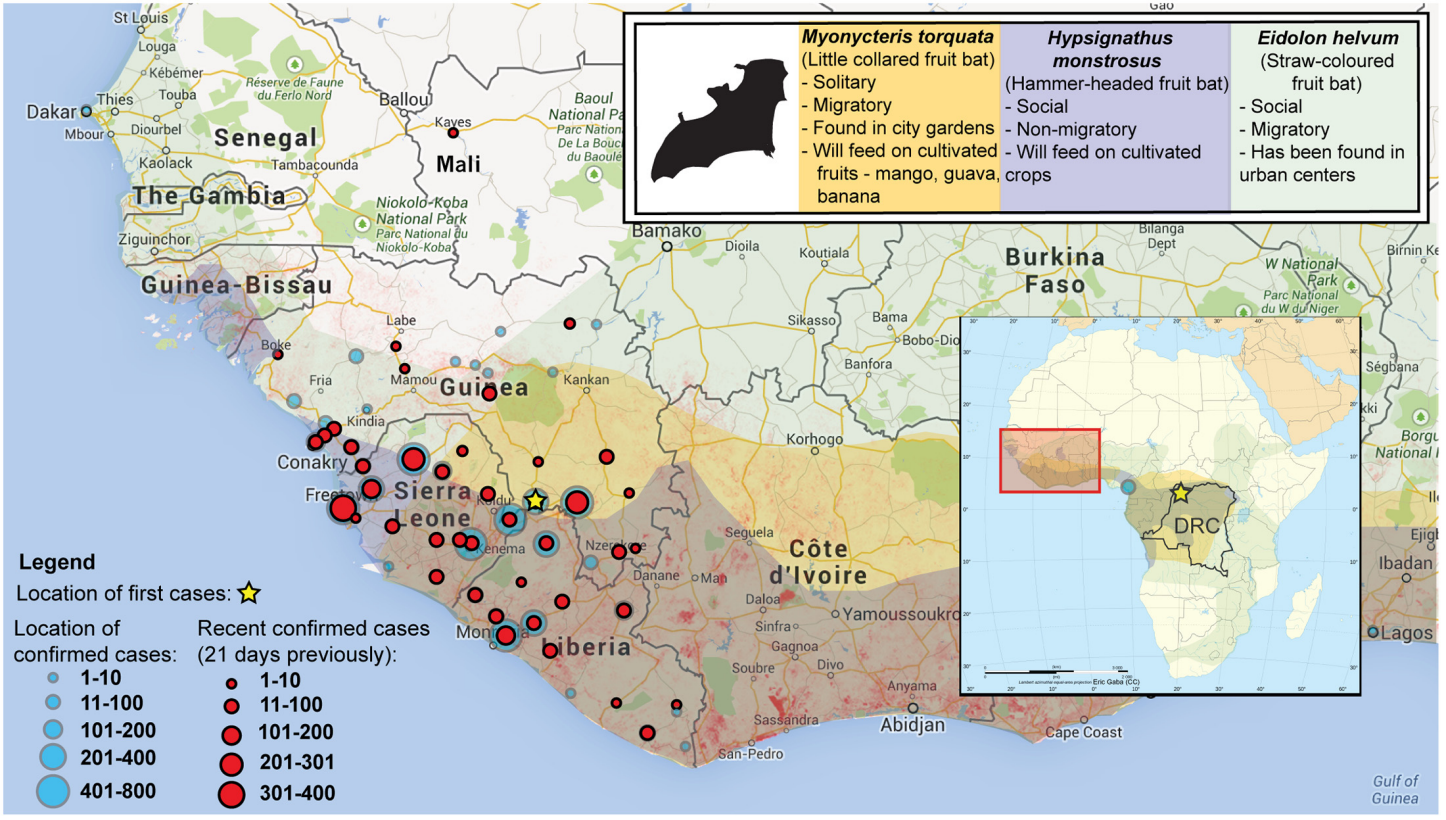
Range of bat species suspected of being reservoirs of Ebola, human population density, and Ebola case counts by location in West Africa. The range of putative EBOV reservoir species the little collared fruit bat (yellow), the hammer-headed fruit bat (blue), and the straw-coloured fruit bat (green) are thought to be associated with previous Central African EBOV outbreaks [40–42]. Guéckédou, Guinea, was the first affected area in December of 2013 (star) [12] with spread to other regions (blue—location of confirmed, red—recent confirmed cases as of October 20, 2014 [43]. The outbreak now involves Sierra Leone and Liberia. Limited spread, in Nigeria and Senegal (only one case), related to travel of infected persons has been identified. A separate Ebola outbreak in the DRC was reported on August 25, 2014 (map inset) [44]. Human-mediated loss of forest resources (2000–2012, red stippling) has been dramatic in the region [45]. In addition to bushmeat-associated exposure, human-mediated environmental change in the region could increase human contact with potentially infected bat species in both the urban and rural environment.

A second outbreak of Ebola was discovered in the rural Boende region of the DRC in August 2014 (Figs 1 and 5). The index case was identified as a pregnant woman who handled bushmeat. Subsequent infections in the community stemmed from contact with the woman’s body during funeral rituals [38]. Phylogenetic analysis confirmed it to be a different strain, unrelated to the 2014 West African outbreak, indicating that a separate zoonotic introduction was responsible for viral emergence into the DRC population [39]. This virus strain is most closely related to virus isolated from the 1995 Ebola outbreak that occurred in Kikwit, DRC. As of October 21, 2014, the outbreak had grown to 67 cases and 49 deaths [38].

## Pathogen Spillover

Spillover of EBOV from the wildlife reservoir to human populations appears to be a complex process involving a number of coupled networks and seasonal drivers (Fig 2) [46], linking the human host to virus reservoirs. Several bat species are considered to be putative EBOV reservoirs, three of which have been a focus of attention with respect to the current West African outbreak: the hammer-headed fruit bat (*Hypsignathus monstrosus*), the little collared fruit bat (*Myonycteris torquata*), and the straw-coloured fruit bat (*Eidolon helvum*) [41,42]. Only frugivorous and insectivorous bat species have shown virus replication and developed high circulating virus titers without showing EBOV-associated illness [47]. Virus found in lung tissues and feces indicates that respiratory, oral, and fecal transmission pathways may all be possible exposure routes to susceptible hosts. Outbreak range overlap is identified in a number of bat species in which EBOV antibodies have previously been found (Fig 5). Some bat species, such as the straw-coloured fruit bat, the largest ranging bats species in Africa, have the ability to migrate long distances (up to 2,500 km) [48]. Thus, movement of EBOV through bat colonies from Central Africa into West Africa would be possible. Alternatively, the virus may have been in the reservoir host for some time, but conditions for spillover did not occur previously.

EBOV transmission to wildlife species (e.g., duiker, nonhuman primates) is thought to occur with ingestion of fruit that has been contaminated with infected fruit bat saliva or feces [41]. Chimpanzees, however, are different and in addition to consuming fruit, will actively engage in predation of other wildlife and nonhuman primates, hunting cooperatively and sharing meat among their social group, a behavior rarely observed in other nonhuman primates, with the exception of baboons [49]. In addition, only chimpanzees will carry meat away from the site of predation—in some instances, more than a kilometer. Scavenging of meat from carcasses is, however, rarely identified among any nonhuman primate species. Chimpanzee hunting has previously been linked to Ebola emergence in Côte d'Ivoire, where the hunting and shared consumption of a red colobus monkey was associated with a large outbreak of Ebola among chimpanzees (Fig 2) [14]. This was the first record of “bushmeat” consumption causing an Ebola outbreak in a nonhuman primate population. A large-scale survey of nonhuman primates across Central Africa only found significant serologic evidence of exposure among chimpanzees (12.9%), suggesting that non-lethal infections do occur in nonhuman primates [50]. Seropositive chimpanzees were found broadly throughout forested regions of Central Africa, identifying Ebola viral circulation in areas where human infections have not yet been identified (e.g., Cameroon [50]). Serosurveillance studies among humans in Central Africa have also identified seropositive individuals even in the absence of a history of Ebola infection or residence in an area where an Ebola outbreak occurred [51]. Understanding spillover in humans continues to be a challenging issue, given the relative infrequency of these events. Important similarities exist in both the physiology and behavior of chimpanzees and humans. Focused research on chimpanzees might provide important insight into Ebola spillover pathways arising from hunting and consumption of bushmeat.

### What is the role of nonhuman primates in virus circulation?

Ebola is a rapidly fatal disease for nonhuman primates [52]. Although a potential source of infection for humans through consumption of dead apes, nonhuman primates are not considered to be a reservoir host or a host species able to maintain sustained viral transmission independent of contact with the reservoir host [52]. Indeed, outbreak mortality in chimpanzees and gorillas has been extreme with some outbreaks, pushing these species closer to extinction [9]. The 2002–2003 epidemic of Ebola in gorillas in the Lossi Sanctuary in northwest Republic of Congo killed 90%–95% of the population, an estimated 5,000 animals [53].

### Seasonal triggers of Ebola outbreaks

Meteorological factors have been associated with a variety of infectious diseases and can have complex influence over contact networks and disease transmission pathways [54]. This is particularly true when wildlife reservoirs are involved in pathogen spillover to other wildlife species and humans. Local and regional weather patterns act as a strong determinant of floral characteristics and surface water attributes within a given landscape. The nature and distribution of these resources can dynamically influence animal behavior, as well as species distribution, species fitness, migration patterns, and population density. These attendant effects can have a profound impact on contact probabilities between susceptible and infected hosts within and between species, as well as the potential for pathogen transmission and spillover to humans [55]. Additionally, contact probabilities can be altered by deforestation and land-use change, which may compound the impact of meteorological phenomena and further cluster susceptible and infected hosts around more limited resources.

**Fig 6:**
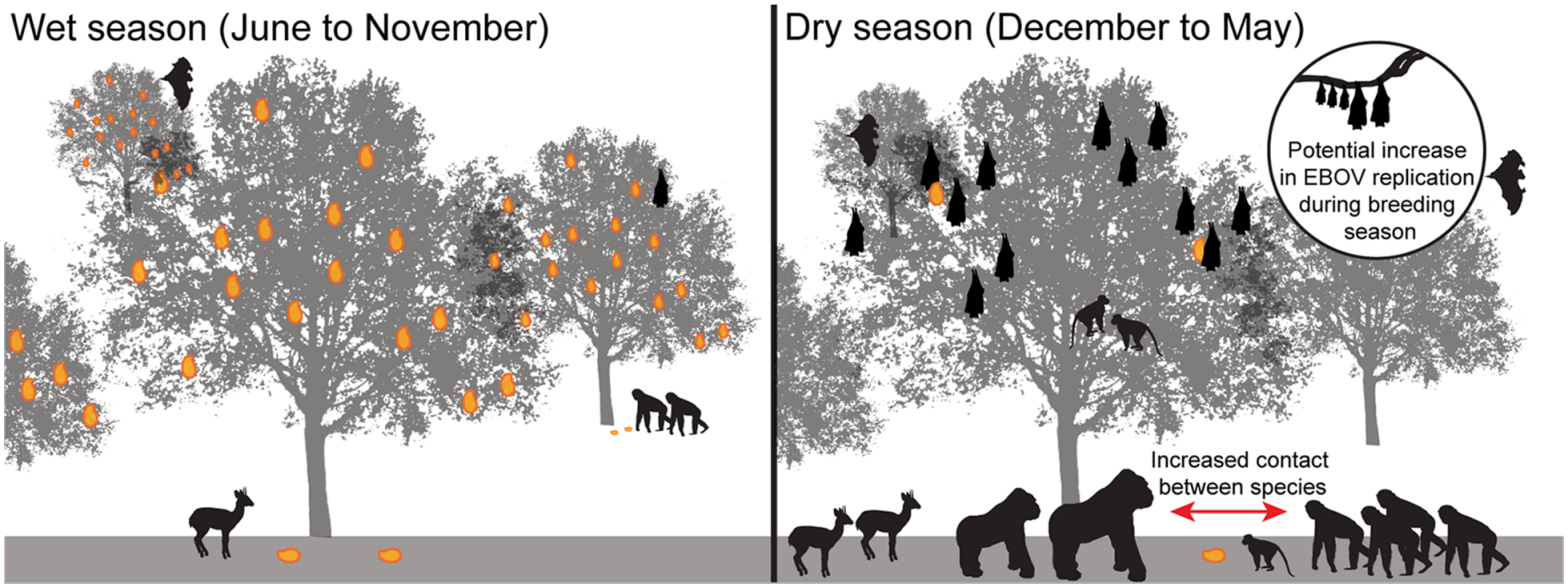
Seasonal factors may influences forage and wildlife distributions, potentially increasing their contact with Ebola reservoirs. Ebola outbreaks appear to coincide with seasonal factors, which can influence forage availability and spatial distribution across the landscape, potentially increasing contact between wildlife species and EBOV transmission potential. Fighting and breeding among bat species during these periods is thought to potentially influence viral load and EBOV transmission within and between bat species.

There has been little analysis of the meteorological and hydrologic conditions associated with Ebola outbreaks. Study of these processes has been hampered by the limited availability of meteorological station observations in Central Africa. A few studies have attempted to circumvent this issue through use of satellite estimates of land surface greenness. In this fashion, Pinzon et al. (2004) examined eight Ebola outbreaks during 1994–2002 and found an association with drier-than-normal conditions at the end of the rainy season. Certainly, hydrologic changes could influence forest fruit production and other resources. Foraging behavior in frugivorous species (e.g., fruit bats, duikers, and nonhuman primates) can be strongly influenced by seasonally driven temporal and spatial clustering of scarce fruit resources [56], potentially concentrating reservoir and susceptible host species in these areas of increased foraging opportunity (Fig 6). A recent study identifies the potential zoonotic transmission niche as a region that covers more than 22 countries in Central and West Africa. These areas are defined by characteristic vegetation, elevation, temperature, evapotranspiration, and range of suspected bat reservoirs [57].

### Human-mediated landscape alteration—Increased contact with EBOV reservoirs?

In the outbreak zone, human-mediated environmental change has been significant, potentially contributing to the emergence of EBOV. The Guinean forest surrounding the outbreak areas is considered a major biodiversity hotspot, containing an estimated one-quarter of all African mammalian fauna [58]. Human encroachment into these areas has been dramatic, with cumulative forest loss estimated to be between 83%–86% (Fig 5) [59]. The landscape is now dominated by forest-agricultural mosaics [58]. These environmental changes in the outbreak region may provide the opportunity for direct exposure to infected bats, potentially creating transmission pathways that do not rely on exposure to bushmeat. For example, the little collared fruit bat can be found in forest/grassland mosaics, an increasing feature of the landscape, and has been identified feeding on guavas and mangoes [60], as well as occurring in more urban areas such as city gardens [61]. Straw-coloured bats have also been identified in human-modified environments, including city parks [62]. The hammer-headed fruit bat can be found in a wide range of habitats, including agricultural areas, where they have been recorded feeding on cultivated crops [63]. The two-year-old child who is the index case in the West African outbreak is assumed to have been exposed by eating bushmeat [10]. However, the child could well have been exposed to bat-contaminated fruit or other bat excretions within the home environment where EBOV-infected bats may occur [41]—a more likely exposure route than eating bushmeat if, indeed, the two-year-old was the first case. It will be important to determine whether Ebola spillover can occur independently of bushmeat utilization and exposure.

## Social Conditions Enabling and Enhancing Human-to-Human Transmission

War, population growth, poverty, and poor health infrastructure, among other social conditions in the outbreak region, have likely contributed to the unprecedented expanse, duration, and size of the EBOV epidemic in West Africa (Table 3). In this region of Africa, population growth has been dramatic, with population densities (people/km^2^) increasing by 223%, 178%, and 275% in Guinea (1960–2012), Sierra Leone, and Liberia, respectively (1961–2013, Fig 7A) [64]. Rural-to-urban migration and growth in the affected countries has significantly increased the proportion of people living in urban environments, where EBOV outbreaks have focused in West Africa. The proportion of the population that is now urbanized has increased significantly in Guinea (248%, 1960–2013), Sierra Leone, and Liberia (130% and 163% respectively, 1960–2013, Fig 7B) [64].

**Table 3:** Socioeconomic and environmental factors may have influenced Ebola emergence in Guinea, Liberia, and Sierra Leone [64].

	Country	Guinea	Liberia	Sierra Leone
**Environmental features**	Country size	94,926 sq miles (245,857 km^2^)	43,000 sq miles (111,370 km^2^)	27,699 sq miles (71,740 km^2^)
	Crop production index increase (2004–2006 = 100) (1961–2012)	246%	118%	388%
	Livestock production index increase (2004–2006 = 100) (1961–2012)	346%	305%	328%
**Human resources and infrastructure**	Number of physicians (per 1,000 people in 2010)	0.1	0.01	0.02
	Improved sanitation (Total, Rural, Urban)	19%, 11%, 33%	17%, 6%, 28%	13%, 7%, 23%
	Improved water source (% of population without access in 2012)	25%	25%	40%
**Population features**	Urban population increase (% of population (1960–2013)	223% increase (1960–2012)	275% increase (1961–2013)	178% increase (1961–2013)
	Historical civil unrest	Yes	Yes	Yes
	Literacy (% of people age 15 and above)	25% in 2010	43% in 2008	44% in 2012
**Cultural and behavioral features**	Use of traditional healers	High	High	High
	Use of traditional burial practices	High	High	High
	Bushmeat consumption	High	High	High

**Fig 7:**
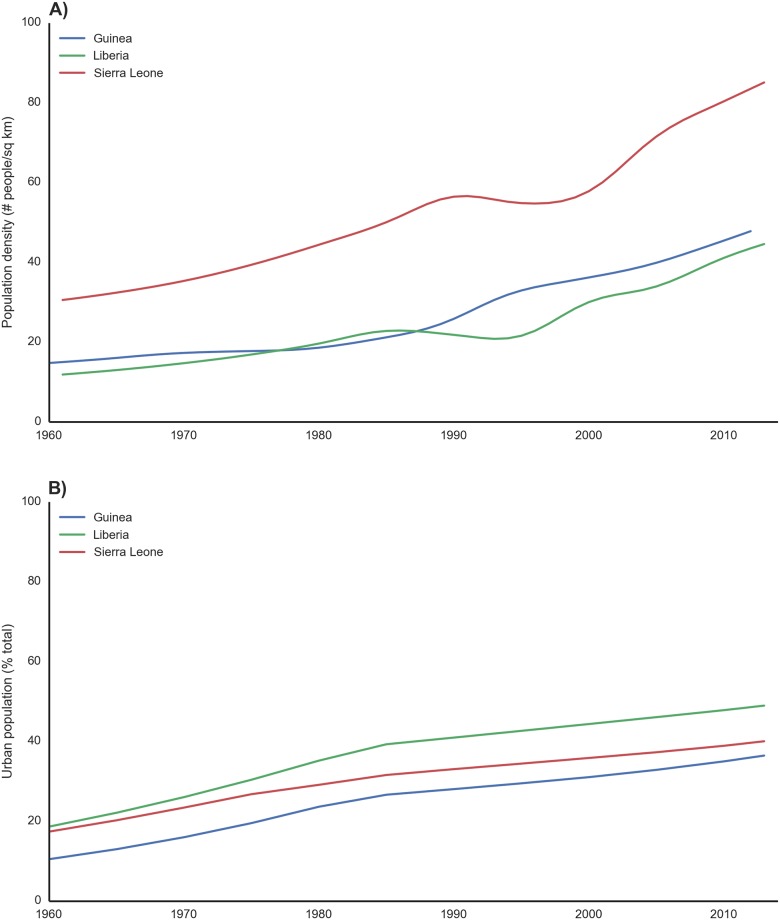
Increases in population density and the proportion living in urban environments in Guinea, Liberia, and Sierra Leone since the 1960s. A) Population density in the outbreak region has increased dramatically over the last 40 years [64]. Increases in human density can have a critical influence on contact networks and human-to-human transmission potential and environmental degradation. Increasing need for natural resources can potentially increase contact rates with wildlife (e.g., timber). B) Urbanization is an important factor influencing infrastructural needs, resources, and population density, factors that can influence contact networks, outbreak dynamics, and intervention success. This is particularly true in poorer countries, where rapidly progressing disease outbreaks in urban environments outstrip weak public health resources. Liberia has experienced the greatest increase in urban population, with an estimated 253% increase since 1961.

### Human mobility

A complex suite of sociological and economic factors influence human movement across the landscape and can have critical impacts on outbreak dynamics and the spatial spread of infectious disease [65]. In West Africa, human movement is considered a particular characteristic of the region [66], with migration rates exceeding movement in the rest of the world by more than 7-fold [67]. An estimated 11% of West African people live outside their country of birth, with between 30%–40% of people residing outside their district or village of birth [68]. In Liberia, for example, 54% of the population over the age of 14 are identified as being internally displaced [69]. Large-scale population movements in the region, both within and between countries, have been driven by decades of conflict and the search for improved socioeconomic conditions and opportunities, identifying an important part of regional livelihood strategies for the poor [68]. As such, present-day population mobility in West Africa has been an important contributing factor to the explosive nature of the West African Ebola outbreak.

The location and nature of the index case and spillover event has also been important to the rapid spread of the epidemic. In this case, the index cluster of infections occurred in Guéckédou, Guinea, a small village bordering Sierra Leone and Liberia near major road networks [1,2]. Infected individuals moved rapidly from the originally infected village into other locations, eventually leading to human introduction of EBOV into major urban centers, such as the capital city of Liberia, Monrovia (mid-June 2014) [70]. Regional expansion of the outbreak to Senegal and Nigeria was associated with travel from affected regions. Fear of rapid Ebola spread across the continent and globe has precipitated border controls on movement to and from the affected countries [71]. Border controls themselves, however, can have important negative impacts on the outbreak, preventing movement of urgently needed supplies and resources, prompting the United Nations Security Council to call for an end to the isolation of affected countries [71].

### Decades of civil unrest

From 1989 to 2004, sustained armed conflict raged in West Africa, moving across borders among Liberia, Sierra Leone, Guinea, and Côte d’Ivoire. Violence, looting, and pillaging became an economic opportunity for impoverished people, and a large mercenary force developed in the region [72]. Mass refugee movements and resettlement camps created a large group of displaced and vulnerable people, with the associated environmental impacts that persist today [73]. These regional environmental and societal disturbances have impacted infrastructure, governance, social cohesion, and the mental and physical health and livelihoods of people in the region [74,75]. These effects have also severely undermined societal resiliency as well as public health infrastructure and service delivery in the region [75,76].

### Behavioral and cultural practices

Consideration of behavior and culture in disease transmission is critical to control and understanding transmission dynamics [77]. Cultural diversity shapes African nations between and within countries and can have a profound influence on social cohesion and communication, particularly during times of disturbance. For example, Liberia has at least 16 major ethnic and cultural groups, each described by a specific language and associated dialects, religion, traditions, and customs [78]. EBOV, because of its nature of transmission, is particularly influenced by cultural and behavioral practices that occur at the household and community levels and within a hospital setting (patient care, family involvement and role, health-seeking behaviors and responses). Consequently, there is no one “community,” and the cultural diversity that defines the region will need to be considered in local disease emergence prevention as well as in the public health response.

### Bushmeat consumption

Bushmeat utilization has been identified as the primary mechanism of EBOV spillover from wildlife reservoirs to humans. Rapid human migration to urban centers has placed increased pressure on the region for food production [58], including access to bushmeat, a preferred protein source [79]. In Liberia, timber extraction, opening of road networks, and influx of worker settlements has been linked to unprecedented increases in bushmeat extraction from forested regions [80]. Bushmeat in Liberia is a critical source of protein, estimated to account for three-quarters of the country’s meat use [81]. In Brazaville, Republic of Congo, 88% of households interviewed reported consuming bushmeat (*n* = 1,050), preferentially mammals (artiodactyls [48.3%], rodents [28.3%], and primates [13.0%]) [82]. Bushmeat has become an important commercial commodity, trafficked illegally both domestically and internationally, potentially providing a mechanism for pathogen spread. Indeed, it has been estimated that approximately 5 tons of bushmeat are illegally imported into Europe each week [83], and it is a common form of contraband moved within and between African nations [84]. While the Ebola virus is susceptible to a variety of disinfectants and can be inactivated by cooking (60°C for 60 minutes) or boiling for five minutes [85], the virus can survive over three weeks at low temperatures in the absence of disinfection or inactivation [86]. This is consistent with epidemiologic data, which identified disease in game hunters [87–89], with none documented in individuals who ate the game after cooking [87]. Wildlife biltong, a dried-meat delicacy that is widely consumed in Africa and abroad, may pose special challenges [90], given that the virus can survive over 50 days when dried and kept at 4°C [86]. At present there have been no confirmed cases of Ebola related to the consumption of dried or smoked meat. However, there is still the concern that movement of biltong could increase the infection risk of wildlife products well beyond the point of animal slaughter to distant markets, given virus survival potential. Cultural practices can also differ importantly as to what wildlife species are used, obtained, processed, and consumed, potentially influencing Ebola transmission risk [77].

### Burial practices

Traditional burial practices, involving washing and touching of the deceased, have been linked to 60% of Ebola cases in Guinea [91]. Caregiving, primarily by women, has also been associated with outbreaks, presumably explaining the relatively high rate of infection in women (67% of affected individuals) in the 2000–2001 Ugandan outbreak [92]. When a traditional healer fell ill with Ebola in Uganda, many individuals from the community came to care for her, and when she died, they took part in her burial [92]. The infected individuals were all women. Spread of the present outbreak into Sierra Leone was also associated with infection and death of a traditional healer and the women who had participated in her funeral [37]. It is important to note that burial practices can be divergent even within a nation, giving rise to the need to consider ethnic diversity and cultural differences within and between villages, towns, nations, and regions and their influence on funeral practices and pathogen transmission dynamics. There is a need to identify more refined data on these activities so that appropriate regionally and culturally specific public health practices can be developed. These data will also aid efforts to model epidemic dynamics; as funerals are an important feature of transmission, the nature of them will define epidemic spread.

### Traditional medicine and cures

Traditional medicine is defined as the total knowledge base, skills, and associated practices that arise from theories, beliefs, and experiences identified by different cultures and used in the maintenance of health. Traditional medicine constitutes the world’s oldest health care, and it has involved the development of culturally and geographically specific techniques for preventing illnesses and diagnosing and treating individuals and communities for centuries. While modern health care based on Western medicine is now considered the norm in many countries, much of Western Africa still relies heavily on traditional practices. Indeed, in countries surrounding the outbreak zone, such as Cote d'Ivoire and Ghana, 70% of the population depend solely on traditional medicine, while in Burkina Faso and the DRC, this figure increases to 80% of the population [93]. While traditional medicine can have a positive role in health care, ethnomedical beliefs can also have important impacts on health-seeking behavior, health outcomes, and pathogen transmission pathways.

Individuals often look to traditional healers and family members for advice and care despite inexperience of the person providing information [94]. Traditional healers may have positions of influence within the community and, therefore, command a level of trust, and can also have a significant influence on health-seeking behavior and uptake of health messages, factors that can directly affect outbreak dynamics. Sick individuals have often opted to listen to traditional healers and rumors about potential “cures,” for example the use of saltwater baths and drinks that have led to recent deaths in Nigeria [95]. Drinking bleach was also considered a way to rid oneself of Ebola in the Ugandan outbreak of 2000–2001 [96]. In the 2005 Ebola outbreak in the Congo, traditional healers declared that cursed “dishonest hunters” caused the outbreak, and many believed this to be true [97]. False information of this sort can significantly affect outbreak dynamics and increase the length and severity of epidemics. Encouragingly, the head of traditional healers in one district of Sierra Leone has recently stopped treating patients, acknowledging that he knows very little about the virus, and called on other healers to suspend healing activities until they are given adequate training [98]. Training traditional healers in infection control and delivery of public health messages might be an important mechanism for the dissemination of information to local communities and reduction in Ebola transmission risk.

### Fear and obstruction of health interventions

Immense fear and anxiety exists toward modern health care providers in Ebola outbreak countries. This fear has stopped many individuals from seeking health care, causing them to instead hide from authorities and revert to traditional healers or family members for care [91]. Sick individuals already admitted to health care facilities have also fled, fearing they will only die in the hospital environment [91]. For example, in the Ugandan outbreak, people feared that once they went to hospital they would never see their families again [92]. In a rural setting, these influences will be important, but in high-density communities, they can be catastrophic in their effect on outbreak dynamics and control efforts. While health care and aid workers have the very best of intentions, the nature and severity of the virus means that quick action must be taken, resulting in the breakdown of communication between patients, relatives, and workers, and the inability of traditional practices to take place, propagating more fear and distrust between the parties. This outcome stems in large part from a lack of understanding and familiarity with Western medicine and practices [94], whereas community values often prioritize traditional practices and consultation and see both as a critical step in any community process engendering trust. For example, with the immediate need to disinfect and dispose of infected corpses, health care workers carried out burials before notifying families [92]. In 1995, during the Kikwit epidemic, all deceased individuals were buried in individual or common graves by the Red Cross staff. The body of one individual, however, was forcibly taken from the hospital to the family’s home to have a traditional burial [99]. The removal of this body led to another (and the final) surge of Ebola infections in Kikwit [99].

Fear is not limited to community members, but is also common among health care workers [96]. These concerns are not unwarranted, as hospital staff are at an increased risk of exposure [100]. Health care worker infection can be catastrophic, particularly where large populations are served by an inadequate public health sector. By September 2014, in the West African outbreak, 10% of the deceased were believed to be health care workers [26]. In the Kikwit outbreak in 1995, 25% of Ebola cases were health care workers, and many left their jobs out of fear of contracting the disease [96,101]. Understaffing of hospitals involved in Ebola outbreaks has led to staff working longer and harder, resulting in exhaustion and an increased potential for deadly mistakes.

### Stigmatization and implications to outbreak containment efforts

Health stigmas can influence the behavior of both the infected and the uninfected during an epidemic, introducing barriers to outbreak management and potentially influencing pathogen transmission and spread, as well as disrupting social cohesion. The AIDS pandemic provided important insight into the critical impacts health-related stigma and social hostility can have on epidemic control measures, highlighting the absolute need to consider these elements in public health strategy development and response [102]. Ebola has perhaps provided a more extreme example. Health care workers, critical to outbreak management, have been harshly stigmatized during Ebola outbreaks, rejected by their communities and families, and even stoned by community members, as they were believed to act as a reservoir for the virus [99]. These same beliefs and stigmas have impacted health-seeking behavior, with the fear of contracting Ebola from health care workers influencing the decision to seek medical help [103]. Fear of stigmatization can influence disease reporting, with victims and their families failing to notify authorities of possible infection because of the potential negative response of their neighbors and community [104]. Ebola survivors can also be heavily stigmatized—many survivors are rejected by their communities, have their belongings burned, and are not allowed to share common amenities [92]. Data from the 2001 Uganda outbreak suggest female survivors experienced more stigmatization than male survivors [92]. Stigmatization can reach beyond the immediate family, as for example in Uganda, where relatives of survivors and the deceased were also stigmatized once the names were publicly released [96]. Fear of stigmatization is not necessarily limited to the level of the individual, household, or community, but may also extend to the level of country governance in which concerns over international response may influence reporting of health information [105]. Health-related stigma has been a prominent feature of the outbreak in West Africa [104] and likely a contributor to the difficulties identified in containing the epidemic.

Health education is one of the keys to combating many issues surrounding Ebola outbreaks, including trust of health officials, the use of non-traditional burial practices, and the acceptance of survivors, relatives of the deceased, and health care workers back into their communities. Health education was seen as one of the major factors in stopping the DRC Ebola outbreak in 1995 [101], and along with contact-tracing and quarantine in the Congo (1995) and Uganda (2000) outbreaks, health education was believed to decrease the effective reproductive rate of Ebola and reduce the final epidemic size by a factor of 2 [106]. However, as important as it is to develop and share health messages, the messages must engage the culture and traditions of the target group or risk having no effect or, worse, a negative effect.

## Ebola Forecasting, Detection Control, Education, and Future Needs

Containment of the West African Ebola outbreak is the most pressing, immediate need. This effort will require mobilization of many additional resources, including medical personnel, educators’ supplies, food, water, and other essential needs. Additional issues need to be addressed to prepare other countries for the possibility of Ebola importation or emergence. Below, we highlight example recommendations that might support enhanced country-level preparedness in Africa and elsewhere, while recognizing that many of these recommendations may be very difficult to implement in the West African countries currently combatting Ebola.

**Fig 8:**
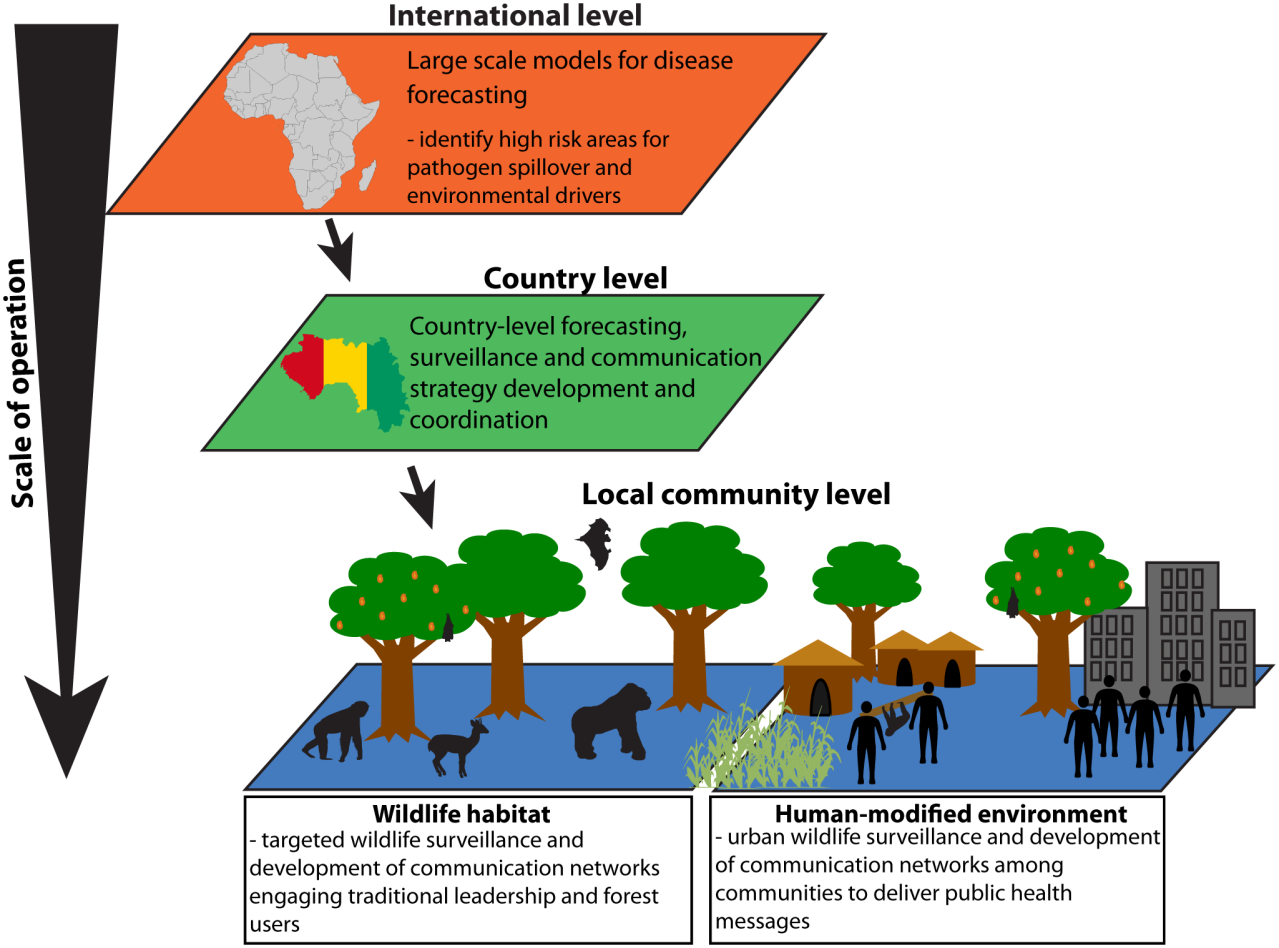
Schematic Ebola early-warning system. Development of any early-warning system for the prevention of future Ebola outbreaks will require a multiscaled effort that spans the international level down to the community, engaging partnerships between and within levels. The most important element of surveillance will be the effective engagement of local communities in regions of concern. A community-driven wildlife surveillance strategy should be designed through participatory approaches, driven by traditional leaders in partnership with country governments. Developed communication networks would need to engage forest users regarding observations of deceased or sick wildlife, in particular those species associated with Ebola outbreaks previously. Sociological assessments and community consultation would be needed to identify barriers to reporting dead or sick wildlife and development of appropriate educational approaches and other social interventions. While international assistance will be important, government and community ownership of the process at the national and local level will be important for sustainability. Research into Ebola reservoir and transmission dynamics will be essential to refining surveillance approaches.

1. **Partnerships and coordinated outbreak response:** Coordinated development of communication strategies and surveillance partnerships across the region will be needed. Governments outside the outbreak region will need to be actively included and assisted as needed to develop national detection and response strategies and protocols. Regional meetings of Health Ministers (e.g., 2nd Extra Ordinary Ministers of Health Meeting on Ebola Virus Disease in Victoria Falls, Zimbabwe, September 4–5, 2014) provide important venues for bringing scientists and policymakers together to ensure that frontline countries have access to the resources they need to manage potential spread and outbreak response. Developing sustained partnerships across Africa and the international community will be critical for our ability to contain this and future epidemics.
2. **Outbreak response in resource-poor settings:** Information collection and communication will still be a challenge in resource-poor settings, and specific strategies will need to be developed to allow rapid identification and response within the context and constraints identified in the local environment. Integrated approaches involving both human and animal health must be developed that engage the research, law enforcement, and policy environments within these local settings.
3. **Human movement:** While protocols have been developed to isolate and test any people displaying signs of illness at cross-border crossings (e.g., http://www.cdc.gov/vhf/ebola/hcp/index.html), protocols are also needed to manage illegal immigrant investigation and holding protocols. These individuals may not have travel documents indicating country visitation or citizenship. Management of these immigrants is often undertaken by multiple agencies (immigration, police, and defense forces). Appropriate training and procedures must be identified to address the multiagency nature of the activity and to allow for safe and respectful management of such individuals during times of heightened concern over human mobility and EBOV spread. Controls on borders must be done securely but in a manner that allows movement of critical supplies to affected regions.
4. **Need for continuing molecular epidemiological outbreak assessments:** Access to samples from the current outbreak is challenging, given the already-impossible burden placed on health staff active in the outbreak site. However, any samples and/or DNA sequence data available should be made accessible to the public health community as soon as possible in order to allow molecular investigations to advance. This will facilitate refinement of our understanding of transmission pathways (e.g., through determining transmission networks) and public health implications, among other areas of need. This information is urgently needed to address the challenge of containing this current outbreak and identifying appropriate control measures.
5. **Modelling tools and data gaps:** Modelling may provide essential information on potential scenarios for outbreak progression, intervention design, and logistics planning [107]. Data gaps in the outbreak region have been significant, however, limiting the full use of this tool set and our ability to address operational needs. Funding priorities for Ebola and other health research in Africa should be outcome-oriented and directed at addressing identified data gaps that are key to prevention and control in order to address immediate needs. Agent-based approaches can incorporate complex cultural and behavioral norms and can be used to direct data collection in data-poor environments [108]. Social media data are often used in public health, both in tracking infections and in delivery of health messages [109,110]. Lack of internet access in the current outbreak requires innovative approaches that will allow bridging of these essential data gaps and delivery opportunities for health messages. Two types of modelling efforts will be important, one that engages emergent needs in an outbreak and a second directed at understanding broader elements of the epidemic and preventing future outbreaks. The scope and focus of each are complementary and allow scalable assessments of outbreak needs, both present and future.
6. **Bushmeat movement and use within Africa—increasing our ability to prevent spillover:** Wildlife smuggling and bushmeat trafficking occur extensively regionally and internationally. Wildlife meat is often deboned and skinned to decrease the likelihood of detection, and can be mistakenly identified as livestock meat. Protocols need to be developed for the safe seizure of suspected or known wildlife products at border crossings or elsewhere in country. Patterns of illegal bushmeat trafficking within and between African countries should be a priority area of investigation and areas of increased risk identified as best as possible for purposes of future outbreak prevention.
7. **Multiscale early-warning systems and future preparedness strategies:** While international and regional modelling efforts provide important tools for forecasting risk zones, community-based surveillance will be necessary to effectively identify Ebola emergence in wildlife (detection of death and/or sickness) before outbreaks occur at the local level (Fig 8). Public health education will be important in reducing behaviors that increase risk of spillover from wildlife sources.
8. **Global Public Health Education Needs:** Public health education regarding Ebola dynamics and transmission is not only needed urgently in Africa but, increasingly, around the world. In the US, public panic appears to be escalating, and there is the risk that choices may be driven by fear rather than fact [31]. A focused program of communication from public health officials is urgently needed and should involve multiple outlets such as radio, television, and social media platforms. These communications should provide factual information concerning the management of Ebola risk, tailored to the target population.

Africa is a changing landscape, and our approaches will have to engage the complexities of the region and community livelihoods. It is clear that many factors could have contributed to the emergence of Ebola in West Africa. Increasing population size, social unrest, and poverty have undoubtedly influenced both the explosive and sustained nature of this epidemic and our collective inability to contain it. We will need to rethink our approach to disease emergence events in low-resource areas, where significant knowledge gaps exist and operational barriers impede isolation and control efforts. The doctors, nurses, public health officials, non-governmental organizations (NGOs), and political leaders are presently challenged with on-the-fly responses to public health emergencies in a low-resource area and are to be congratulated for their ingenuity and perseverance. The real partnerships that are emerging among community leaders, NGOs, governments, and international agencies must be encouraged and facilitated to the greatest possible extent.

**Key Learning Points**

- Significant political, social, and environmental changes have occurred in West Africa, likely contributing to the emergence of the most deadly Ebola outbreak in history.
- Similarity in outbreak characteristics (including R0, symptoms, incubation time, and serial time) between West Africa and previous Ebola outbreaks suggests that there has not been any significant change in the virus affecting transmissibility.
- Information collection and communication remain a challenge in resource-poor settings and specific strategies and tools will need to be developed to allow rapid identification and response within the context and constraints identified in the local environment.
- Integrated approaches involving both human and animal health must be developed that engage the research, law enforcement, and policy environments within these local settings.

**Top Five Papers**

1. Leroy EM, Kumulungui B, Pourrut X, Rouquet P, Hassanin A, Yaba P, et al. Fruit bats as reservoirs of Ebola virus. Nature. 2005;438:575–576.
2. Gire SK, Goba A, Andersen KG, Sealfon RS, Park DJ, Kanneh L, et al. Genomic surveillance elucidates Ebola virus origin and transmission during the 2014 outbreak. Science. 2014;345:1369–1372.
3. Pinzon JE, Wilson JM, Tucker CJ, Arthur R, Jahrling PB, Formenty P. Trigger events: enviroclimatic coupling of Ebola hemorrhagic fever outbreaks. Am J Trop Med Hyg. 2004;71:664–674.
4. Frieden TR, Damon I, Bell BP, Kenyon T, Nichol S. Ebola 2014—New Challenges, New Global Response and Responsibility. N Engl J Med. 2014;371:1177–1180.
5. Rivers CM, Lofgren ET, Marathe M, Eubank S, Lewis BL. Modeling the Impact of Interventions on an Epidemic of Ebola in Sierra Leone and Liberia. 2014; arXiv preprint arXiv:14094607.
